# The Behaviour of Cs, Co, and Sr Radionuclides in Marine Environmental Sediment

**DOI:** 10.1100/tsw.2002.290

**Published:** 2002-06-07

**Authors:** Nariman H.M. Kamel

**Affiliations:** Radiation Protection Department, Nuclear Research Center, Atomic Energy Authority, P. O. Box 13759, Cairo, Egypt

**Keywords:** marine sediment, sorption, desorption, surface complex, sequential extraction

## Abstract

This work describes experimental investigations and modelling studies on the sorption of radionuclides Cs, Co, and Sr by certain marine sediments within Egypt. The chemical composition of the marine sediments was determined. The soluble salts were measured for the sediments and the concentrations of the released cations, Al, Fe, and Si, were measured for the sediment materials in 0.1 M NaClO_4_ aqueous solution at different hydrogen ion concentrations. The two main factors that control the uptake of the radionuclides onto the sediment are the pH and the exchangeable capacities of the sediment materials. Surface complex model was used to estimate the surface charge densities and the electric surface potential of the marine sediment materials. These two parameters were calculated at the surface capacity sites of the sediment materials. The desorption of the adsorbed cations was determined by means of selective consecutive extraction tests using different chemical reagents including (1) 1 M MgCl_2_ (pH 7), (2) 1 M ammonium oxalate (pH 3-5), (3) 0.04 M NH_2_OH,HCl in 25% acetic acid (pH 3-4), (4) H_2_O_2_ in 5% HNO_3_(pH 2-3), and (5) digestion with nitric acid followed by hydrofluoric and perchloric acids (pH 2).

## INTRODUCTION

Sediment is a major sink of many kinds of wastes that arise from human activities. Sorption of various kinds of pollutants, including radionuclides such as $^{137}Cs$, $^{134}Cs$, $^{60}Co$, $^{90}Sr$, and $^{85}Sr$, on marine sediment materials is governed by the concentrations and the chemical forms of the contaminant, mineralogical composition of the sediment materials, and the physico-chemical composition of the ecosystem[1].

The sorption process depends on the electrostatic interaction of solute at the surface of the sediment, the chemical reaction of solute with the sorbing surfaces, precipitation of solute on the sediment surface, and metal dissolution of mineral surfaces. These subjects were extensively reviewed in some previous works[2,3]. Several approaches have been used to describe and create a model for the adsorption reactions. Surface complex models are now finding widespread application in the field of pollutant retention[4]. In the surface complexation approach, sorbing ions from solution are considered to react chemically with the functional groups of the solid phase surfaces. Sorption was assumed to take place at specific surface sites having specific sorption capacities. The surface of the sediment may be assumed to be covered with a neutral, negative, and positive charge. These were represented as: $\equiv S\text{-}OH^o$, $\equiv SO^-$, and $\equiv SOH_2^+$. Here "S" means a metal of the mineral surface and "≡" indicates the strong bond between the metal and the other part of the mineral structure. Hence the surface charge may be more negative (positive) as the pH increases (decreases)[3,4,5]. Different experimental approaches have been used to investigate the mobility of metals in environmental solid samples; one of the applications is the selective extraction technique. The samples were treated with a succession of reagents to liberate metals with different affinities for the matrix. This technique detects fractions of trace elements of various types that are easily desorbed, ion-exchangeable, bound to Mn oxides, bound to iron oxides, bound to organic material, and so on[6,7,8,9]. The objective of this study is to test the applicability of the surface complexation model to the marine sediment material and to study the effect of leaching by different chemical reagents on the retention of the trace elements and $^{134}Cs$, $^{60}Co$, and $^{85}Sr$ radionuclides.

## METHODS

### Marine Sediment

In this work, five bottom sediment samples of various composition were studied. These samples were collected from the sea bottom at a 3- to 4-m depth from four locations near the shore of the Mediterranean Sea and one location near the shore of the Red Sea (see Fig. 1).

The sampling sites are located at:

- Agami, a western suburb of Alexandria (A)
- Damietta and Gamasa, near the east Nile estuary (D & G)
- Port Said, at the north of Suez Canal (P)
- Sharm El-Sheikh (S) between the two north gulfs of the Red Sea

Particle size distributions were carried out by sieving. $CO_3^{2-}$ was determined by back titration[10]. X-ray diffraction as well as the chemical analyses of the major cations was carried out. The powder sediment samples were fused with 30% NaOH aqueous solution and digested with the concentrations of HF and $HClO_4$. The aqueous solution was analyzed for the major cations. In these analyses, Al and Fe were determined by spectrophotometer using Ferron (8-Hydroxy-7-iodoquinoline-5-sulphonic-acid)[11]. Na, K, Ca, and Mg were analyzed using Dionex liquid ion chromatography. Si was analyzed using molybdate method[11]. The total organic carbon (TOC) percentage was determined by ignition of 5 to 10 g of the soil at 600°C for 2 h and then cooled at room temperature. The TOC was calculated by the difference in weighing the sediment samples before and after ignition. The specific surface area (SSA) was determined by the ethylene glycol method[10].

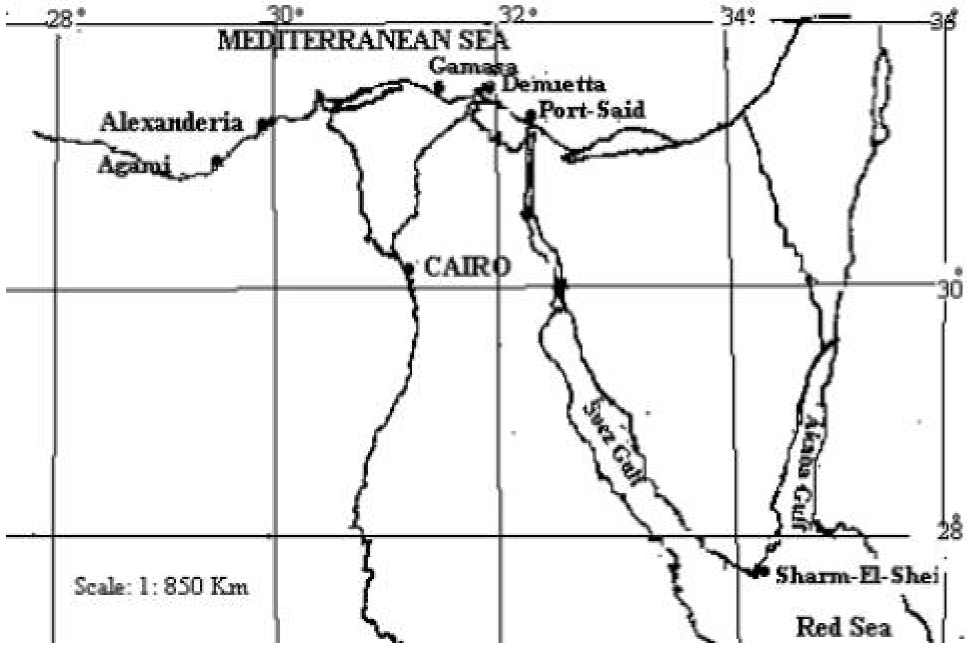

**FIGURE 1.** The site locations of the sediment samples: Agami (A), Damietta (D), Port Said (P), Gamasa (G), and Sharm El-Sheikh (S).

## Soluble Ions and Cation Exchange Capacity

The soluble cations, anions, and alkalinity of the sediment samples in distilled water were determined. The major cations of $Na^+$, $K^+$, $Ca^{2+}$, and $Mg^{2+}$ and anions of $F^-$, $Cl^-$, and $SO_4^{2-}$ were also determined using a Dionex liquid ion chromatography. Alkalinity was determined as $HCO_3^-$ by titration with $H_2SO_4$ acid.

Two experiments were conducted for the determination of the exchangeable ions. In the first experiment, the exchangeable cations were determined using ammonium acetate and glacial acetic acid at the neutral pH 7[10]. In the second experiment, acid/base back titration tests were carried out using the sediment samples in an inert 0.1 mol/l, NaClO4 aqueous solution at the pH (3 to 10). The maximum time allowed for the adjusting of the pH after the addition of acid or base was about 30 min. The final pH of sediment in the aqueous solution as a function of the amount of acid or base adsorbed was measured. Concentrations of Al, Fe, and Si released were determined at different pH in the range 3 to 10.

## Sorption Experiments

Batch sorption experiments were carried out in duplicate. Distilled water was used as a solvent. CsCl prepared as a carrier at the concentration $10^{-6}$ mol/l traced with $^{134}Cs$ radionuclide. $CoCl_2$ prepared as a carrier traced with $^{60}Co$ and $SrCl_2$ prepared as a carrier at the concentration $10^{-3}$ mol/l traced by $^{85}Sr$ radionuclide.

There were 15 ml of various aqueous electrolyte solutions added to a known weight (0.5 to 1.0 g) of the sediment sample in 20-ml polyethylene vials. The suspension was shaken at 40 rpm for 14 days and the aqueous layers were separated from the solid layer by centrifugation at 4,000 rpm for 10 min. The activities of $^{134}Cs$, $^{60}Co$, and $^{85}Sr$ radionuclides in the aqueous phase were measured using gamma spectrometer NaI (TI) crystal. The uptake percentage (U%) was evaluated from the batch sorption experiments using the following formula, where $A_o$ and $A_1$ are the activities of the blank and the aqueous solution:

$$U\ (\%) = \frac{A_o - A_1}{A_o} \times 100 \qquad (1)$$

## Desorption Experiments

Desorption experiments were carried out by replacing the contaminated aqueous layer solution with a certain volume (100 ml) of the different chemical reagents solutions that are shown in Table 1. The solutions were sequentially conducted to leach the following fractions: 1- exchangeable, 2- carbonates, 3- oxides (Fe and Mn), 4- organic and the residue. Concentrations of the radionuclide released by leaching with the different reagent were calculated as the desorption percentage (D%) using the following Eq. 2 to 4:

$$D_1\ (\%) = \frac{A_2}{A_o - A_1} \times 100 \qquad (2)$$

$$D_2\ (\%) = \frac{A_2}{A_o - A_1 + xA_1} \times 100 \qquad (3)$$

$$D_3\ (\%) = \frac{A_2 - xA_1}{A_o - A_1} \times 100 \qquad (4)$$

$A_2$ is the activity of the aqueous solution desorbed from the solid phase, and $x$ is the volume (in ml) of adsorption solution remaining in the bottle after the desorption. The soils were air dried and weighed each time before the next sequence started.

**TABLE 1**
**Sequential Extractions Tests[6,7,8,9]**

| Desired Phase | **Reagent (200 ml)** | **Time of Extraction (h)** |
|---|---|---|
| 1- Water soluble/exchangeable | $H_2O/MgCl_2$ 0.4 mol/l, pH 4.5 | 1 |
| 2- Carbonates | $NH_4COOCH_3$ 1.0 mol/l/25% acetic acid, pH 4 | 2 |
| 3- Oxides (Fe/Mn) | $NH_2OH.HCl$ 0.04 mol/l/25% acetic acid, pH 2 | 5 |
| 4- Organic | $H_2O_2$ 30 % $HNO_3$ 0.02 mol/l, pH 2 | 5 |
| 5- Residue | $HNO_3/HClO_4$ digestion + 30 % NaOH fusion | 0.25 |

**TABLE 2**
**Chemical Analyses and the Grain Size Distributions of the Marine Sediment Samples**

| Oxide % | Sediment Sample | | | | |
|---|---|---|---|---|---|
| | A | D | P | G | S |
| $SiO_2$ | 68.5 | 77 | 64.2 | 72.8 | 68.5 |
| $Al_2O_3$ | 17.4 | 18.9 | 20.41 | 15.12 | 20.03 |
| $Fe_2O_3$ | 1.7 | 1.4 | 5.7 | 4.3 | 2.9 |
| $Na_2O$ | 2.2 | 0.3 | 0.03 | 1.13 | 2.4 |
| $K_2O$ | 1.4 | 0.96 | 2.3 | 0.38 | 0.36 |
| CaO | 3.5 | 1.4 | 0.7 | 3.8 | 1.4 |
| MgO | 2.2 | 0.76 | 0.17 | 0.05 | 0.32 |
| $H_2O$ | 3.1 | nil | 6.5 | 2.5 | 4.1 |
| Organic carbon (%) | 0.001 | 0.001 | 0.0021 | 0.002 | 0.002 |
| Sum (%) | 100.00 | 100.72 | 100.12 | 100.08 | 100.01 |
| **Grain size (μm) %** | **Sediment sample** | | | | |
| | **A** | **D** | **P** | **G** | **S** |
| Fine sand >50 - >200 | 50.6 | 81.32 | 48.1 | 65 | 81.19 |
| Silt >2 - >50 | 48.4 | 17.17 | 38.6 | 33 | 17.7 |
| Silt + clay >5 - ≥2 | 1 | 1.51 | 13.3 | 2 | 1.11 |

## RESULTS AND DISCUSSION

Table 2 shows the results of the chemical analyses and the grain particle size distribution (≥ 200 to ≥ 2 μm) of the fine sand, silt, and silt with clay fractions mixed with the clay minerals. Fig. 2 shows the spectra of X-ray diffraction of the marine sediment samples. The mineralogical composition of the sediment samples under investigation indicated that the samples consist of the major quartz, calcite, and kaolinite minerals mixed with the moderate illite and feldspar minerals. The sediment sample Agami (A) consists mainly of the calcite mixed with the moderate portion of kaolinite minerals. The sediment sample of Damietta (D) consists of the major kaolinite and quartz minerals. The sediment sample of Port Said (P) consists mainly of amounts of kaolinite mineral mixed with a moderate amount of illite mineral and minor quartz mineral. The sediment sample of Gamasa (G) consists of muscovite mixed with minor amounts of illite, quartz, and kaolinite minerals. The sediment sample of Sharm El-Sheikh (S) consists of quartz as a major consistent mixed with the moderate calcite and feldspar minerals.

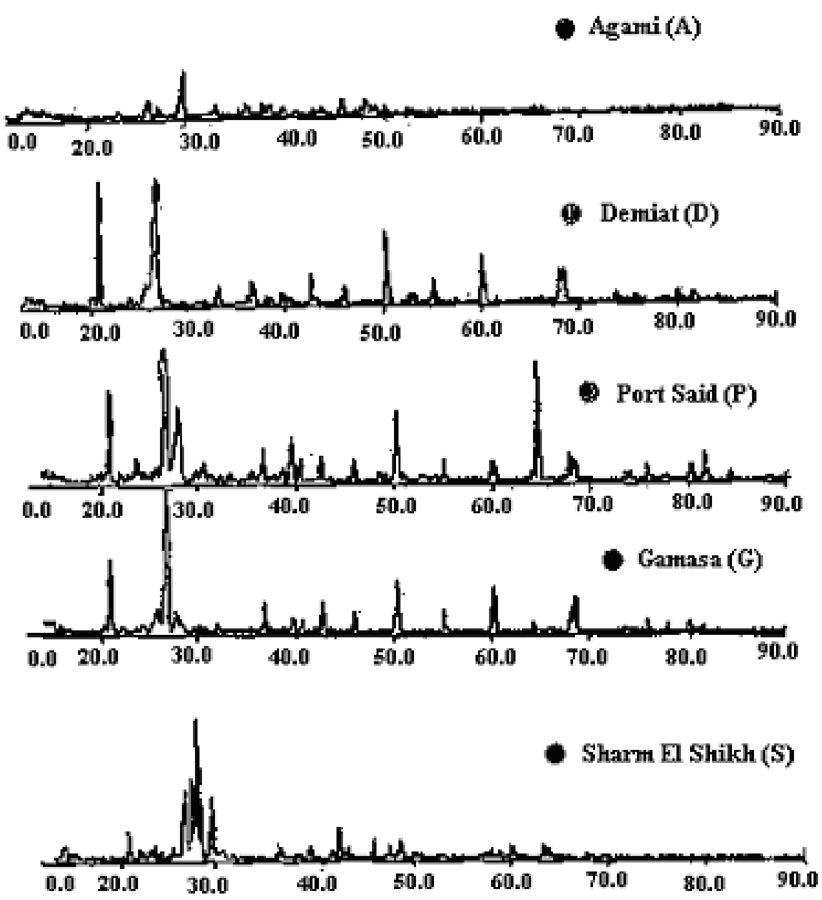

**FIGURE 2**. X-ray defraction of the marine sediment samples. Agami (A), Damietta (D), Port Said (P), Gamasa (G), and Sharm El-Sheikh (S).

## Soluble and Exchangeable Ions

The major soluble cations and anions released with distilled water are shown in Table 3. The sediment sample A has less soluble salts 6.2 meq/100 g; out of these, the soluble Na is 4.35

**TABLE 3**
**The Soluble Cations and Anions of the Marine Sediment Samples**

| Element Released from Distilled Water (meq/100 g) | Sediment Sample | | | | |
|---|---|---|---|---|---|
| | A | D | P | G | S |
| Na | 4.35 | 21.22 | 7.13 | 8.70 | 8.87 |
| K | 0.05 | 0.80 | 0.35 | 0.31 | 0.100 |
| Mg | 0.86 | 1.91 | 0.43 | 1.37 | 1.32 |
| Ca | 0.95 | 1.70 | 1.80 | 1.90 | 1.90 |
| Sum cations (meq/100 g) | 6.20 | 25.63 | 9.71 | 12.27 | 12.18 |
| F | 0.04 | 0.05 | 0.05 | 0.04 | 0.08 |
| Cl | 4.43 | 22.29 | 9.77 | 9.77 | 11.20 |
| $SO_4$ | 1.21 | 0.72 | 0.85 | 1.29 | 1.13 |
| Alkalinity ($HCO_3^-$) | 0.01 | 0.01 | 0.004 | 0.001 | 0.001 |
| Sum (anions + alkalinity) | 6.68 | 24.05 | 11.08 | 11.20 | 12.51 |
| Standard error (%) | 3.7 | 3.2 | 6.59 | 4.54 | 1.32 |

**TABLE 4**
**The Cation Exchange Capacity of the Marine Sediment Samples**

| Cation Released from Ammonium Acetate (meq/100 g) | Sediment Sample | | | | |
|---|---|---|---|---|---|
| | A | D | P | G | S |
| Na | 8.59 | 10.13 | 15.11 | 16.52 | 7.10 |
| K | 0.25 | 1.19 | 1.25 | 0.69 | 0.25 |
| Mg | 2.32 | 4.32 | 0.32 | 0.31 | 2.26 |
| Ca | 10.73 | 3.12 | 0.95 | 0.62 | 6.86 |
| Sum CEC (meq/100 g) | 21.88 | 15.64 | 16.44 | 16.44 | 16.44 |
| Carbonates (%) | 19.7 | 8.2 | 4.0 | 2.0 | 13.0 |
| The correct value of the CEC (meq/100 g) | 2.18 | 7.44 | 12.44 | 14.44 | 3.44 |

meq/100 g and the low soluble K is 0.05 meq/100 g. The sediment sample D has a high soluble salts of 24 meq/100 g; out of these, it has a high soluble Na of 21.22 and K of 0.8 meq/100 g. A slight difference of the soluble salts was found for the sediment samples P, G, and S (10 to 12 meq/100 g). The cations released were found to decrease in the order $Na^+ > Ca^{++} > Mg^{++}$ and $K^+$. The anions released were found to decrease in the order $Cl^- > SO_4 > F^-$. Concentrations of $Na^+$ and $Ca^{++}$ represent nearly 88 to 94% of the sum of the released cations. Concentrations of $Cl^-$ and $SO_4^{--}$ represent 81 to 93% of the sum of the released anions. Therefore the majority of $Na^+$ and/or $Ca^{++}$ in the marine sediment samples may be present as chloride and/or sulphate. The rest of the cations may be represented as carbonates or bicarbonates. The CEC (meq/100 g), calculated by the sum of the released cations, $Na^+$, $K^+$, $Ca^{++}$ and $Mg^{++}$, are given in Table 4. It was found that their values range between 16 and 22 meq/100 g. The corrected values of the CEC were calculated from the sum of the released cations minus alkalinity expressed as carbonates. Determination of the CEC of the sediment samples (see Table 4) show that the CEC are sensitive to the mineralogical structure of the sediment samples. The X-ray data are given in Fig. 2 show that the CEC of the sediment samples have the codes; A and S are 2.2 and 3.4 meq/100 g. These low CEC of the kaolinite clay mineral are in agreement with other studies that indicate the kaolinite clay mineral has layers bound more tightly together and that permit less substitution of other ions[12]. The other sediments have the codes D, P, and G and have the CEC values 7.4, 12.4, and 14.4 meq/100 g, respectively. These sediment samples consist of the minor illite clay mineral. The presence of the minor illite clay mineral is responsible for the increasing of the CEC. The values of the CEC, determined in this work, are in fair agreement with the corresponding values determined in previous works[13]. The difference in the CEC depends on the mineral composition of the sample[14].

## Surface Complex Model

The surface of the sediment material in this model is represented by the plane of the hydroxyl groups S-OH, where S represents solid of the sediment material that contains various elements such as, Al, Fe, Mn, and Si or other elements on the surface.

The solid surfaces of the sediment were assumed to be electrically neutral. It was covered with the similar sites of the OH groups. Hence, the negative charge of the OH groups on the solid surface sites tends to attract cations. At the low pH, the $H^+$ ion concentrations react with the negative charge sites and on the surfaces of the sediment as follows:

$$\equiv SOH^o + H^+ \Leftrightarrow SOH^+_2 \qquad (5)$$

and at the high pH, the $OH^-$ ions react with the surface sites of the sediment, according to

$$\equiv SOH^o + OH^- \Leftrightarrow SO^- + H_2O \qquad (6)$$

Due to the change of the pH of the sediment in solution, some metals such as Fe, Al, and Si were released from the sediment particles to the aqueous phase. At equilibrium, the surface of the solid sediment may be considered covered with $\equiv SOH^o$, $\equiv SOH_2^+$, and $SO^-$ groups. The net surface charge density is given by:[5]

$$\sigma_p = \frac{F}{S}\left[\langle \Gamma H^+ \rangle - \langle \Gamma OH^- \rangle\right] \qquad (7)$$

where $\sigma_p$ is the surface charge density (coulombs /$m^2$); $\Gamma H^+ - \Gamma OH^-$ are the amount of $H^+$ and $OH^-$ ions consumed (mol/kg); F is the Faraday constant (96,490 coulombs/mol); S is the specific surface area ($m^2$/kg); $\langle \ \rangle$ sorption density of ion in (mol/kg).

The surface charge density at the inner surfaces of the sediment will be diffused into the sediment materials. The diffused charge layer, according to Gouy-Chapman, at 25°C is given by[4,5]:

$$\sigma_d = 0.1174.\ C^{1/2}\ .\ \sinh (Z\psi.\ 19.46) \qquad (8)$$

where $\rho_d$ is the surface diffuse charge density (coulombs/$m^2$); C is the molar electrolyte concentration (mol/l); Z is the valence of electrolyte; $\psi$ is the surface potential (Volts).

At equilibrium, the overall electrical neutrality, the charge due to the surface layer and the charge due to the diffuse layer canceled each other. Hence: $\sigma_p = - \sigma_d$. Thus the surface potential $\psi$ could be calculated according to Eq. 8.

## Application of the Surface Charge Density

Fig. 3 (A-S) shows the titration curves of the hydrogen and/or hydroxyl ion adsorbed as a function of the pH. The surface site capacities of the sediment samples A, D, G, P, and S were calculated from the titration curves. Table 5 shows the CEC of the sediment samples that were calculated from the titration curves. The sediment samples A and D gave one value of the capacity at the high pH 8 to 9. Since these sediment samples contain high $CO_3^{--}$, some dissolution of the carbonate minerals at the low pH is expected, which led to equal surface site capacities at the pH values 8 to 9 on the surfaces of the sediment materials. The other sediment samples D, S, and G have two sites of the sorption capacities: one at the low pH 4 to 5 and the other at 8 to 9. From these results, it is clear that the sorption capacities depend on the mineralogical composition of the sediment samples. Similar results were observed by other investigators using acid-base titration on Na-montmorillonite in 0.01 M $HClO_4$ aqueous solution[15,16]. These authors showed similar capacities as a result of an acid-base titration on Na-montmorillonite and explained the results on the consumption of the protons by the surface sites of S-OH[15,16]. The SSA of the investigated sediment samples are given in Table 5. The surface charge density ($\sigma$) and the

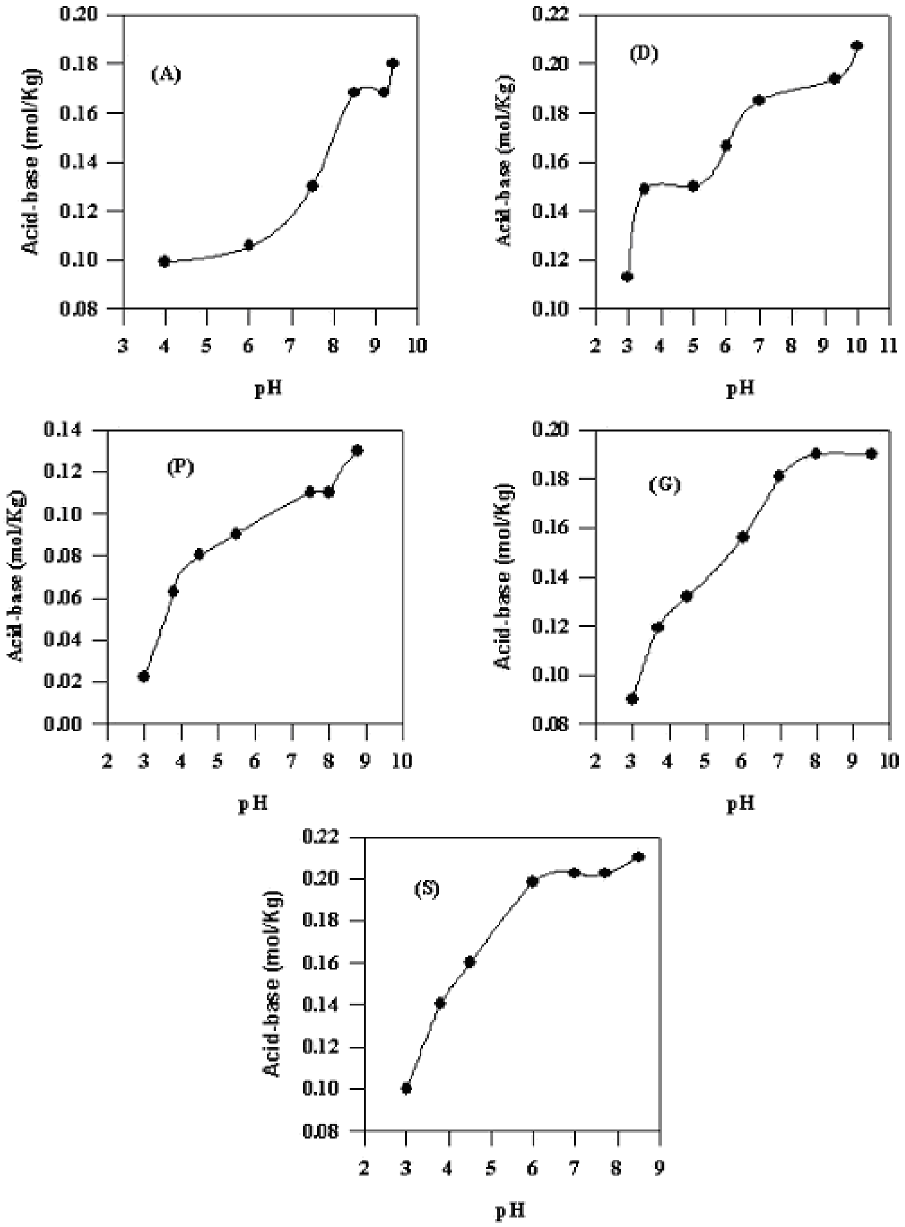

**FIGURE 3**. Acid-base titration of the sediment samples A-S at 0.1 mol/l $HClO_4$ aqueous solution.

electric surface potential ($\psi$) for the marine samples were calculated using Eq. 7 and 8. The obtained results were found to be more or less similar to that previously reported[15,16].

The concentrations of Fe, Al, and Si that dissolved in the aqueous phases at different pH are given in Table 6. Concentrations of Fe and Si were found to increase and decrease by increasing of the pH respectively. The release of Fe and Si were found to be dependent on the mineralogical composition of the sediment sample. No Al was released at the different pH for the sediment samples A and D. For the other samples, the concentrations of Al released were found to increase from 11 to 40 mol/l by the increasing of the pH from 3 to 10.

**TABLE 5**
**The Sorption Capacities, SSA, Charge Densities, and the Surface Potential of the Marine Sediment Samples**

| Sample | PH | CEC × $10^{-3}$ (mol/kg) | SSA ($m^2$/kg) | σ ($m^2$/C) | Ψ (volts) |
|---|---|---|---|---|---|
| A | 8-9 | 0.165 | 67 | 0.24 | 0.131 |
| D | 4-5 | 0.15 | 55 | 0.26 | 0.136 |
| D | 7-9 | 0.19 | 47 | 0.31 | 0.144 |
| P | 4-5 | 0.09 | 60 | 0.31 | 0.144 |
| P | 7-9 | 0.11 | 60 | 0.18 | 0.116 |
| S | 6-9 | 0.20 | 59 | 0.33 | 0.147 |
| G | 4-5 | 0.13 | 71 | 0.18 | 0.116 |
| G | 7-9 | 0.19 | 81 | 0.23 | 0.128 |

**TABLE 6**
**Si, Al, and Fe Released from the Sediment Samples (A-S) at Different pH**

| Sample | A | D | P | S | G | A | D | P | S | G | A | D | P | S | G |
|---|---|---|---|---|---|---|---|---|---|---|---|---|---|---|---|
| | Fe × $10^{-6}$ mol/l | | | | | Al × $10^{-5}$ mol/l | | | | | Si × $10^{-6}$ mol/l | | | | |
| PH | | | | | | | | | | | | | | | |
| 3 | 4.8 | 2.3 | 0.09 | 1.6 | 2 | 0 | 11 | 0 | 12 | 0 | 12 | 19 | 7.5 | 19 | 12 |
| 3.5 | 4.8 | 3.9 | 0.01 | 2.3 | 3 | 0 | 13 | 0 | 12 | 0 | 13 | 19 | 12 | 19 | 13 |
| 4 | 1.8 | 1.4 | 0.01 | 2.0 | 1 | 0 | 19 | 0 | 14 | 0 | 7.9 | 18 | 9.3 | 18 | 8 |
| 5 | 1.6 | 1.8 | 1.1 | 0.07 | 1 | 0 | 11 | 0 | 12 | 0 | 5.9 | 18 | 9.3 | 11 | 6 |
| 6 | 1.6 | 2 | 1.8 | 0.05 | 1 | 0 | 28 | 0 | 25 | 0 | 3.8 | 11 | 11 | 6 | 4 |
| 7 | 2.1 | 2.3 | 1.7 | 0.05 | 1 | 0 | 30 | 0 | 31 | 0 | 4.3 | 5.1 | 11 | 6 | 4 |
| 8 | 2.7 | 2.1 | 11.6 | 0.06 | 1 | 0 | 30 | 0 | 29 | 0 | 4.3 | 5.1 | 9 | 6 | 4.3 |
| 10 | 2.7 | 3.0 | 12 | 0.06 | 1 | 0 | 30 | 0 | 39 | 0 | 4.3 | 6 | 6 | 8 | 4 |

## Surface Adsorption–Desorption of Cations

Fig. 4 shows $^{134}$Cs, $^{60}$Co, and $^{85}$Sr radionuclides released percentage using various chemical reagents (Table 1, procedures 1 to 5). The released percentage of the exchangeable fraction was found to be in the range of 12 to 18% for $^{134}$Cs radionuclide, 2 to 6% for $^{60}$Co, and 24 to 58% for $^{85}$Sr. The released percentage for the carbonate fractions was found to be 10 to 12% for $^{134}$Cs, 4 to 8% for $^{60}$Co, and 17.5 to 43% for $^{85}$Sr. The released percentage for the reducible fractions such as iron was found to be in the range of 16.5 to 22% for $^{134}$Cs, 7 to 19% for $^{60}$Co, and 0.5 to 6% for $^{85}$Sr. The released percentage for the acid soluble fraction was found to be in the range 39 to 51% for $^{134}$Cs, 48 to 70% for $^{60}$Co, and 0.5 to 29% for $^{85}$Sr. The residue percentage for $^{134}$Cs was found to be in the range of 4.4 to 14.4%, 1.8 to 11.2% for $^{60}$Co-60, and 0.4 to 4% for $^{85}$Sr.

Therefore, the exchangeable fractions released percentage was found to decrease in the following order: Sr > Cs > Co. The carbonate fractions released percentage was found to decrease

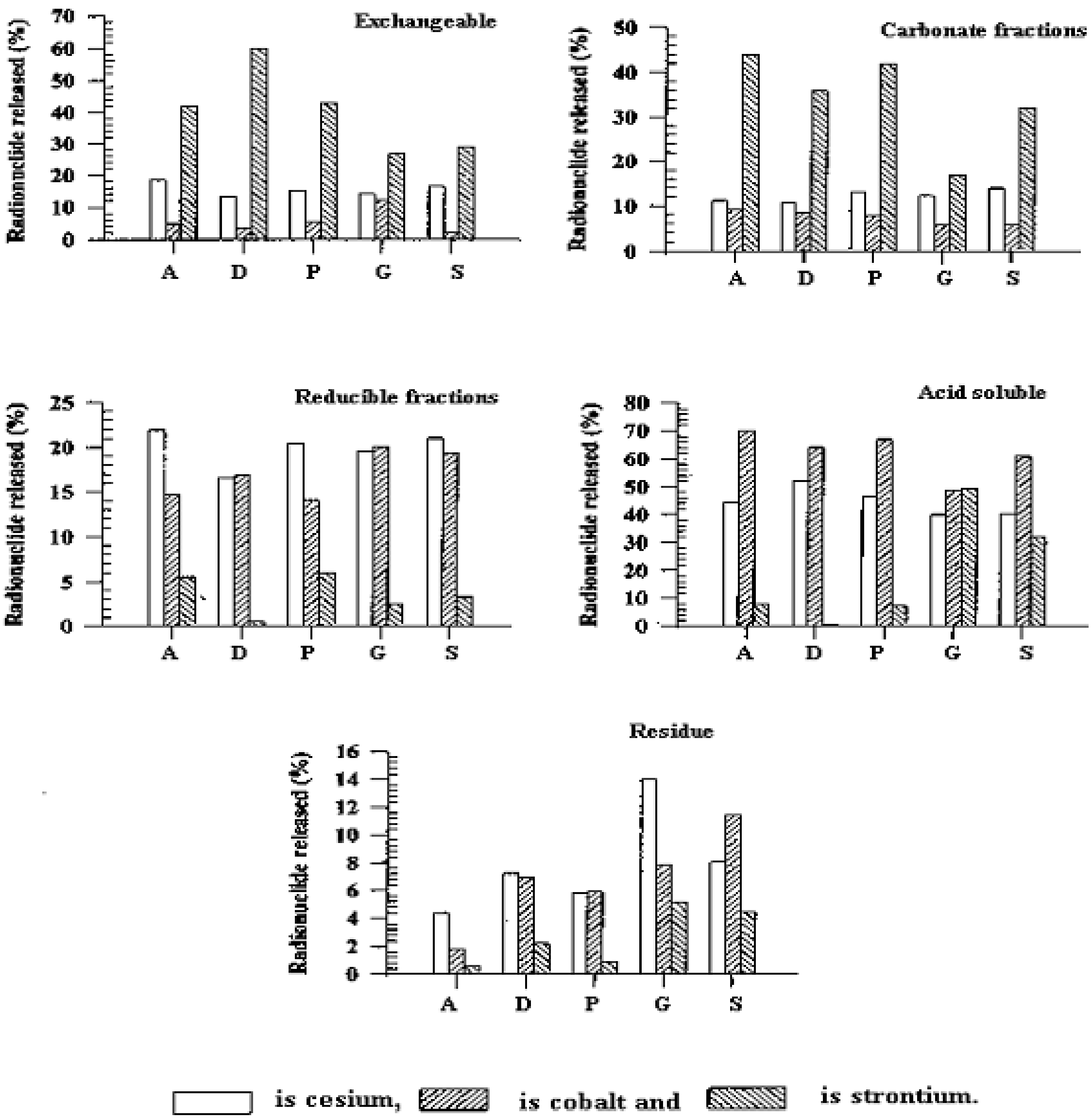

**FIGURE 4**. $^{134}$Cs, $^{60}$Co, and $^{85}$Sr radionuclides released percentage by different sequential extractions for the following: exchangeable, carbonates, reducible, acid soluble, and the residue (see legend): open box = cesium, ///// box = cobalt, \\\\\ box = strontium. A, D, P, G, and S are the Sample Code of Agami, Dameitta, Port Said, Gamasa, and Sharm El-Sheikh.

in the following order: Sr > Cs > Co. The reducible fractions released percentage was found to decrease in the following order: Cs > Co > Sr. The acid soluble fractions released percentage was found to be Co > Cs > Sr. The residue fraction was found with the following order Cs > Co > Sr. These results indicate that the release of Cs and Co radionuclides are affected by the mineralogical structure and acids. These results are compared with that observed by other investigators[17] for the release of $^{137}$Cs and $^{60}$Co by the marine sediments. The behaviour of $^{134}$Cs was attributed to nuclide diffusion into the solid matrix. The behaviour of $^{60}$Co is the combined effects of diffusion and development of bond between the nuclide and the adsorption sites[17].

## CONCLUSIONS

The sorption of $^{134}$Cs, $^{85}$Sr, and $^{60}$Co on marine sediment in Egypt was investigated. The CEC was calculated by the sum of the exchangeable cations and by acid/base titration technique. The results of the acid-base back titration and the SSA of the sediment samples were used for the determination of the charge surface density and the electrical potential of the sediment samples. Selective chemical extractions were used to determine the various fractions that attached to the adsorbed radionuclide. The activity of radiocesium-released percentages was similar to the activity of the $^{60}$Co radionuclide that were attached to the exchangeable and carbonate fractions. $^{85}$Sr release (in percentages) was found to be lower than the $^{134}$Cs and $^{60}$Co release (in percentages) from various fractions. The release of the radionuclides was affected by Fe content in the sediment sample. The residue of the sequential extractions was found to be nearly 10 to 14% in $^{134}$Cs and $^{60}$Co and less than 5% in the residue of $^{85}$Sr release.

## ACKNOWLEDGEMENTS

I would like to thank Prof. H. F. Aly for his valuable comments and constructive changes to this paper. I would like to thank the IAEA for supporting me and allowing me to participate and present this paper in the conference in Mar del Plata. I would like to thank the referees for the suggestions and the valuable comments.
